# Bibliometric Study of Technology and Occupational Health in Healthcare Sector: A Worldwide Trend to the Future

**DOI:** 10.3390/ijerph17186732

**Published:** 2020-09-16

**Authors:** Esther Vaquero-Álvarez, Antonio Cubero-Atienza, Pilar Ruiz-Martínez, Manuel Vaquero-Abellán, María Dolores Redel Mecías, Pilar Aparicio-Martínez

**Affiliations:** 1 SRH Kliniken Landkreis Sigmaringen, Hohenzollernstraße 40, 72488 Sigmaringen, Germany; esther.vaquero@srh.de; 2 Departamento Ingeniería Rural, Ed Leonardo da Vinci, Campus de Rabanales, Universidad de Córdoba, 14071 Córdoba, Spain; ajcubero@uco.es (A.C.-A.); ig1remam@uco.es (M.D.R.M.); 3 GC24 Clinical and Molecular Microbiology, Instituto Maimónides, Facultad Medicina y Enfermería, Campus de Menéndez Pidal, Universidad de Córdoba, 14071 Córdoba, Spain; mi1rumap@uco.es; 4 GC12 Clinical and Epidemiological Research in Primary Care, Instituto Maimónides, Campus de Menéndez Pidal, Universidad de Córdoba, 14071 Córdoba, Spain; en1vaabm@uco.es; 5 Departamento de Enfermería, Fisioterapia y Farmacología, Universidad de Córdoba, Campus de Menéndez Pidal, 14071 Córdoba, Spain

**Keywords:** healthcare workers, ICTs, occupational health, scientometric analysis

## Abstract

Since the eighties, technological tools have modified how people interact in their environment. At the same time, occupational safety and health measures have been widely applied. The European Agency for Safety and Health at Work considers that information and communication technologies are the main methods to achieve the goals proposed to improve working life and the dissemination of good practices. The principal objective was to determine the trends of publications focused on these technologies and occupational safety in the healthcare sector during the last 30 years. A bibliometric study was carried out. The 1021 documents showed an increased trend per country, especially for the United States (*p* < 0.001) and year (*p* < 0.001). The citations per year showed significant differences between citations of articles published before 2007 (*p* < 0.001). The year was also linked to the increase or decrease of articles (72.2%) and reviews (14.9%) (*p* < 0.001). The analysis of journal co-citations also showed that the main journals (such as Infection Control and Hospital Epidemiology) were linked to other important journals and had a major part in the clusters formed. All these findings were discussed in the manuscript and conclusions were drawn.

## 1. Introduction

Since the eighties, technological tools have modified how people interact with their environment [1]. At the same time, occupational safety and health (OSH) measures were widely applied due to the recommendation of the World Health Organization (WHO) regarding the health and working environment [2]. Both changes facilitated the inclusion of information and communication technologies (ICTs) in the working environment [3]. Numerous countries, such as the United States (US) or the United Kingdom (UK), included information and communication technologies (ICTs) in several structures from industries to healthcare systems for workers and consumers [4,5,6].

Following the technological growth in the last decade, multiple ICTs, digital and analog substructure, gears, and widgets [7] have been created to improve the health of the populations and control risks in the environment [8,9]. In this sense, one of the main points for creating ICTs has been the development of different structures to improve the accessibility and sharing of information [9,10,11]. Among these new technologies, smartphones, computers, or tablets have become the favorite tools to access or exchange information [12,13,14]. This preference is based on their ubiquitous, easy-to-use nature and fast features [15]. Nevertheless, the use of ICTs in the workplace could be described as both favorable and unfavorable. Positively, when robotics and other technological advances are effectively used, hazards can be reduced, and training can improve [16]. However, when these technologies are misused, these tools can introduce new dangers and impact the psychosocial health of workers [17,18]. In this sense, several studies have analyzed the influence of ICTs on workers’ health, highlighting mental stress or burnout syndrome, muscular problems and audio-visual alterations, and even addiction [19,20]. Nevertheless, the impact that technology has in any area is partially determined by the creator’s desire or intention, and the impact of ICT depends on the user’s profile and reason for utilizing such technology [21].

Despite the negative effect, European organizations, especially the European Agency for Safety and Health at Work, consider that ICTs are the primary method to achieve the goals proposed to improve working life and disseminate good practices [16]. Nevertheless, the different analyses indicated that the ICTs were not entirely integrated into occupational health and that depending on the sector, the actions of the OSH focused on one area [22,23]. An example would be that financial and scientific sectors focused on psychosocial prevention and health promotion activities, in which ICTs focused on control or training, mainly in industrial environments [24,25]. Meanwhile, the social and health sector was also more likely to promote health in the workplace, with a particular interest in promoting healthy lifestyles [22]. These results are highly contradictory since the health sector workers are more exposed to psychosocial problems, such as burnout syndrome, musculoskeletal disorders, and biological accidents [26,27,28,29]. Different reviews have shown how ICTs, especially websites or databases, could decrease these risks and promote a healthier working environment [30,31]. However, it seems that the latest ICTs created mainly focused on improving structures, surgical approaches, or treatments for patients [32,33]. Even though healthcare workers are at high risk of suffering from a disease or accident, their working environment is up against constant changes and depends on the needs of the population [31,34]. The ICTs could be applied to increase prevention knowledge and skills, changing the environment to improve workers’ well-being and optimize prevention through adequate human and material resources [35]. In fact, previous studies have stated different benefits of ICTs in the occupational health in healthcare sector from continuous training to productivity improvements [36,37]. The most outstanding benefits have been increasing efficiency, reducing errors and improving integration of best practice into routine care [36]. Currently, the ICTs in OHS seem to focus on improving clinical information systems, personal digital assistants or keeping health records of personnel [38]. Nevertheless, the analysis of previous works as a vital step in research is essential [39,40]; therefore, it is imperative to determine the previous knowledge and current tendencies in this scientific field.

Based on this, the principal objective of the current study was to determine the trends of publications focused on ICTs and occupational safety and health in the healthcare sector during the last 30 years (from 1989 to 2019). Additionally, the second objective was to determine the major sub-topics regarding the use of ICTs in occupational safety in the healthcare sector. The purpose of these objectives was to understand ICTs and OSH’s interaction better to assist the decision-making of health professionals and contribute to effective prevention.

## 2. Materials and Methods

### 2.1. Design of the Study

A bibliometric study was carried out to analyze the data regarding the inclusion, use, or implementations of ICTs as an occupational safety measure for healthcare workers. The data focused on the year of publication, country, affiliation, authors id, citations, index keywords, and journal.

### 2.2. Database Selection

Before the use of the research strategy and the analysis of the data, exploratory research was carried out to select the adequate database for the objectives and study proposed. The exploratory research included diverse Medical Subject Heading (MeSH) (“technology” combined with “occupational safety”, “workers”) and several databases (Web of Science, Scopus, PubMed, the Health and Medical Collection, and the Psychology Database). These databases were selected based on their relevance and global use in the health field. The results of the databases were compared to determine the selection of the database for the research strategy. The exclusion criteria were the period of time from 1989 to 2019 and papers with no scientific relevance such as news, obituaries, projects, or patents, available in journals.

The research strategy (ALL = (“technology” AND “occupational safety” AND “workers”)) showed different results for Web of Science (428 documents), Scopus (9479 documents), PubMed (553 documents), the Health and Medical Collection (15,418 documents), and the Psychology Database (14,568 documents). These results displayed a similar number of documents for Web of Sciences and PubMed, higher number of documents for Scopus, and the Health and Medical Collection and Psychology Database had the highest quantity. However, the Health and Medical Collection and Psychology Database also included greater number of grey data than the other three databases (Web of Science, Scopus and PubMed). The significant difference between these databases was the theme and topic of the research, therefore, the main two databases were Web of Sciences and Scopus. The documents obtained using Scopus included the largest abstracts and citations in the field of study. This database has over 16,000 peer-reviewed journals, conference proceedings, trade publications, book series and patents, offering the widest coverage available for scientific, technical, medical and social sciences [41]. Only one database was selected based on the coverage of the topic and the objective, and the fact that previous research indicated how Web of Science and Scopus have high similarities [42]. Based on previous research and the results obtained, it was decided to choose Scopus (Elsevier’s database) since it was the major database focused on the topic [43,44], providing necessary information for the quantitative analysis. Additionally, to avoid Scopus’ problems with the differentiation between authors of the same name, the data have been manually revised and later checked with the authors’ information.

### 2.3. Data Collection

The data were retrieved in June of 2020 from the Scopus database. After obtaining all the data, the information was saved in an Excel sheet, using Excel version 17, with a .csv format, to be later analyzed in SPSS (IBM Corporation, Armonk, NY, USA) program version 25.

Based on an initial analysis, the research strategy included more MeSH terms (Table 1). The main terms were “technology”, “occupational health” and “healthcare personnel”. Other terms, such as “healthcare workers”, related to the MeSH terms (Table 1), were also included in the search to extract more data. The Boolean operators used were “OR” and “AND”, and the fields were “title”, “abstract”, and “keywords”.

### 2.4. Exclusion and Inclusion Criteria

The inclusion criteria used for this study focused on terms related to the topic such as “workers” or “professionals”, “technology”, and “healthcare” to determine the link between ICTs in occupational health. With the results from the terms “occupational health” or “occupational safety” and “education”, it was estimated that the prevention and promotion of a healthy environment were included. Additionally, “mobile” or “mobile applications” terms were used to identify this specific tool, which is more commonly used to determine implications and find previous studies in which the tool or specific applications were developed or analyzed. The Boolean operators used were “OR” and “AND” to link the terms. Additionally, the inclusion criteria regarding the type of document focused on articles, reviews, conference papers, chapters of books, books, editorial and letters. The main language was English, although other languages with their roots based on Latin, such as Spanish, Portuguese, French or Italian, and other Indo-European languages and Uralic languages, i.e., German or Hungarian, were also included.

The exclusion criteria used were the period for the production of the documents (eliminating those documents published before 1989 and after 2019), articles focused on patients, the study focused on workers from other sectors, such as mining, and lack of ICTs’ role. Additionally, the type of document was determined in order to exclude non-scientific productions, such as projects.

### 2.5. Research Strategy

**Figure 1:**
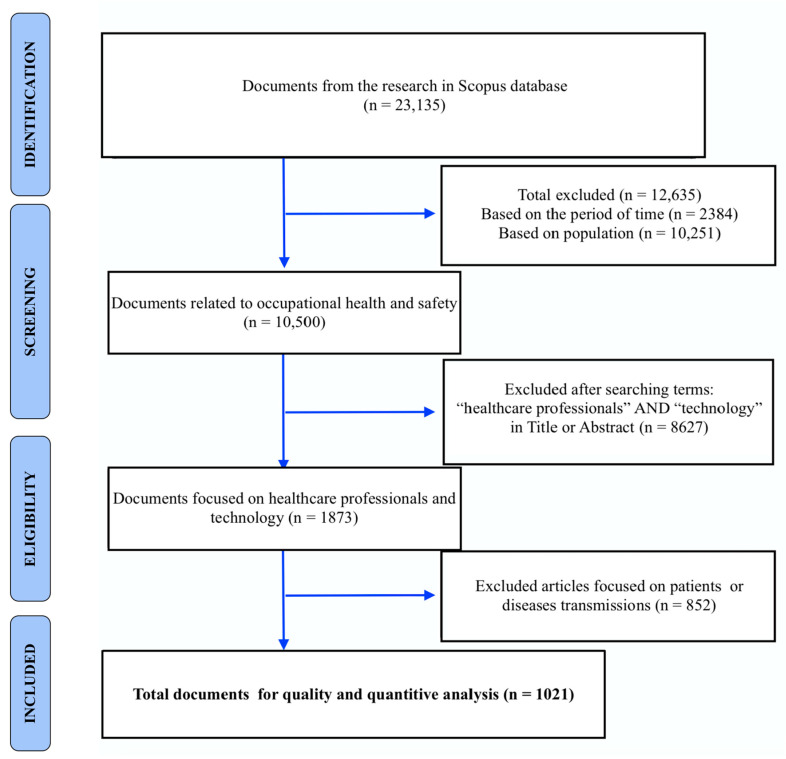
Flow diagram of the selection of articles for the quantitative and qualitative analysis.

The search strategy to gather the data used the different terms and steps (Figure 1), as follows:

1. Identification: in this step, the terms were chosen and the Boolean operators “AND” and “OR” were used. The search query was the following: (TITLE-ABS-KEY (technology OR “social media” OR “Mobile applications” OR “Education, Distance”) AND TITLE-ABS-KEY (“occupational health” OR “occupational safety”) AND TITLE-ABS-KEY (“Health Personnel”)) OR (TITLE-ABS-KEY (technology) OR TITLE-ABS-KEY (“social media”) OR TITLE-ABS-KEY (mobile) OR TITLE-ABS-KEY (“e-learning”) OR ABS (“e-learning”) OR TITLE-ABS-KEY (online)) AND (TITLE-ABS-KEY (health) OR (TITLE-ABS-KEY (“occupational safety”)) OR (TITLE-ABS-KEY (“healthcare workers”))) AND (TITLE-ABS-KEY (prevention) OR TITLE-ABS-KEY (safeguard)). This strategy was developed to identify the number of published items in which title, abstract, or keywords were included the terms (N = 23,135).
2. Screening: A total of 12,635 documents were excluded based on the timeframe and the population of the study, which were workers in other sectors or patients using the filters from the Scopus database. Later on, an additional 8627 documents were eliminated from the data based on their use of keywords in their titles or abstracts.
3. Eligibility: In this step, the abstract or the full document of the remaining 1875 documents were read to determine the main subject of the study. In this step, 852 documents were excluded from the final sample since they focused on disease transmission, such as HIV, or patients’ opinions regarding the worker health or tasks.
4. Included: In this final step, 1021 documents were included for the quantitative and qualitative analysis of the data.

### 2.6. Analysis Technics and Statistical Analysis

The analysis technics used were both quantitative and qualitative. The techniques focused on counting the number of papers linked to countries, institutions and authors, and the counting of citations. Furthermore, the impact of published work on the topic was analyzed using the Journal Citation Report (JCR) and the quartile (Q1) to present the importance and relevance of the major journals. The Journal Citation Report is based on citations compiled from the Science Citation Index Expanded and the Social Sciences Citation Index and the quartiles based on ranking each journal according to their subject, using the impact factor distribution the journal occupies for that subject category as a measure [45]. The citation weighting depends on the subject field and the prestige of the citing serial. The counting of co-occurrence between authors and countries was also determined. Additionally, techniques for visualizing scientific variables (co-occurrence of keywords, countries and authors) were used via VOSviewer [46], an open-source program, as a method of multidimensional analysis. The identification of networks using the VOSviewer software version 1.6.15 [46] was carried out to examine and create bibliometric maps [47]. The networks identified focused on co-occurrence of countries and keywords. Additionally, the co-occurrence of the authors based on the citations was carried out. The criteria used to create the maps were a minimum of five connections between countries and a maximum of fifteen countries per document. The strategy was a minimum of five connections and a maximum of ten for the co-occurrence of keywords, eliminating the term “human/s” to avoid discrepancies. The criteria used for the co-citation was the cited author and journals, with a minimum of 20 citations per author.

The results from the research were analyzed, initially using descriptive analysis, such as the frequencies of documents per country and year, the language, primary sources, the field of the publication, the leading scientific institutions, associations among nations, the primary authors in the area, and the index keywords used. Additionally, the descriptive analysis was carried out focusing on relative frequencies, mean, standard deviation (SD), median and confidence intervals (CI) 95%. To carry out the frequencies and relative analysis, and later statistical analysis, Excel (Microsoft, Redmond, WA, USA) version 2017, Numbers and SPSS (IBM Corporation, Armonk, NY, USA) version 25 were used.

Previous to the statistical analysis, the Kolmogorov–Smirnov test was applied to determine the normalization in the sample. The results of this test (*p* < 0.001) suggested that variables (year of publication, citations or number of documents per country) did not follow a normal distribution in the entire sample. Based on these results, the non-parametric tests (Chi-square, Kruskal–Wallis, and Mann–Whitney U test) and the correlation test (Spearman’s correlation test) were used for the different variables. Additionally, Cramer’s V test was used to determine the magnitude of the differences, such as statistics to determine the size of the effect. The variables analyzed were year of publication, country, language, type of document, keywords, citations, references (co-citations), author’s id, JCR and quartile. The chi-square one test was used to determine if there were differences among the sample for the year of publication, journal, keywords used, language, type of journal and country. Based on the results obtained, the sample was divided and compared by language (English and non-English), country (US and other countries), the type of document (articles compared to reviews; and articles compared to the other documents) and the period of publication (before 2017 and after 2017). The chi-square was used for comparing countries (US and other countries) and language (English and non-English) to having or not having citations. The Mann–Whitney test was used to compare the country (US and other countries) according to the number of publications and citations per country, ranking the countries based on citations and number of documents. The Kruskal–Wallis test was used for the variables: year, citations, JCR and score of the journals. The test associated the different subgroups inside the sample, comparing the year (before 2000, before 2017 and after 2017), the number of citations (less than 100, less than 175 and less than 400) and score of journals using the quartile between the variables and with other variables, such as type of documents ranked based on the number (from articles to conference papers) or languages. The Spearman’s rank correlation coefficient was used to determine associations between the countries, year of publication, language, type of documents and citations.

## 3. Results

### 3.1. Characteristics of Documents: Publication Trend and Language

**Figure 2:**
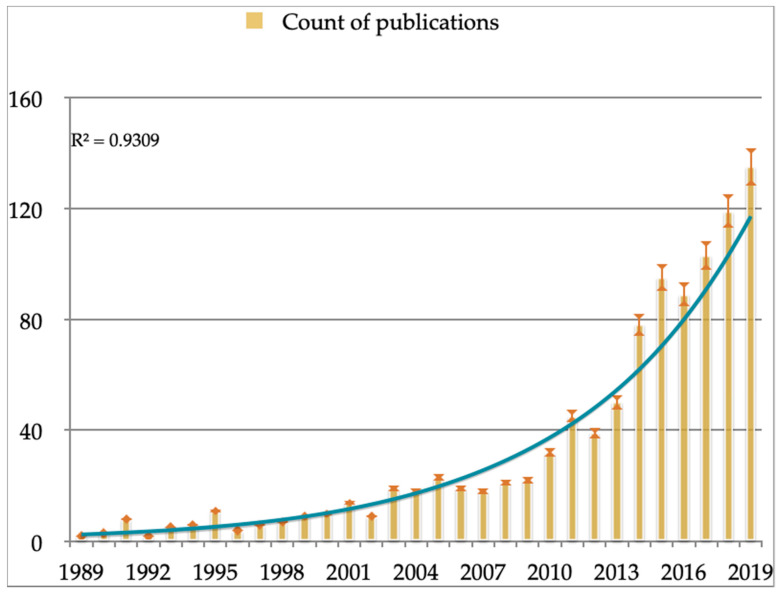
Number of papers per year and exponential trendlines. Note: the error bars and exponential trendline are based on percentage and count of publications.

The 1021 documents, focused on ICTs in occupational safety and health of healthcare workers, showed an exponential trend of publications per year for this timeframe (Figure 2). The frequency of production per year increased from 1989 (0.29%) to 2019 (13.22%), although depending on the years the number and frequency of publication changes. However, the increase has not been linear and there have been some fluctuations, decreasing the number of publications in a time frame (from in 2005, in which 23 documents were published, to 2009, with 22 documents). The exponential trendline (Figure 2) showed that the trend of publications followed a growth at a progressively higher rate, based on R-squared value close to 1. This result showed that the line almost fit the data perfectly. In this sense, the number of documents showed a significant increase from 1989 (two documents published by Kenya and US) to 2019 (135 documents, published mostly by US with 30 documents) (*p* < 0.001). This growth continued to be significant during the timeframe, although there were some modifications in the *p*-value and the grade of the significance (from 2000 to 2019 = *p* < 0.01; and from 2010 to 2019 = *p* < 0.05). Additionally, the trend of publications was analyzed according to the number of citations per year of publication, showing significant differences between documents published before 2017 (mean of documents with citations = 14.35; SD = 24.71) and after 2017 (mean of documents with citations = 0.15.15; SD = 7.02) (*p* < 0.001). The main language of these documents published was English (92.4%), followed by Spanish (1.3%), German (1.0%), French (1.0%) and Italian (0.8%).

### 3.2. International Dissemination of Publications and Collaorations between Countries

Table 2 indicated that most documents were published by the US (with 369 documents), followed by the UK (55 documents), Australia (51 documents), Canada (50 documents), and Germany (55 documents). The mean of publications of the top five countries showed difference between US (mean = 11.9), UK (mean= 1.8), Australia (mean= 1.7), Canada (mean = 1.6) and Germany (mean = 1.2). Furthermore, the Chi-square test for a sample indicated significant differences between countries for the production of documents (*p* < 0.001), with a significant difference between US and other countries. Additionally, Cramer’s V test was used to determine the size of the effect for country and publications according to the years, showing significant differences between countries (*p* < 0.001). However, the value of the test (Cramer’s V= 0.32) was lower than expected, proving less association between the variables of country and production of documents per year.

Table 3 showed how among the top ten publications with more citations, most documents were: published in the US, articles, focused on public health, and published in the first decade of the 21st century. Furthermore, the table showed how most journals were from a medical area, with all of them ranking in the first quartile (Q1) according to journal impact (Journal Citation Report), which were related to the citations (*p* < 0.01). 

The number of citations (mean = 12.4; SD = 21.4) per document was analyzed showing a difference between countries (*p* < 0.001) and year of publication (*p* < 0.001). The top five countries with more documents (Table 2) had significant differences (*p* < 0.01) regarding the number of citations and documents with citations, with the US the leader (mean = 14.9; SD = 30.4; CI 95% = 18.1–11.7), followed by UK (mean = 11.7; SD = 13.4; CI 95% = 15.4–7.9), Australia (mean = 9.7; SD = 13.3; CI 95% = 13.5–5.8), Canada (mean = 8.6; SD = 9.6; CI 95% = 11.4–5.8) and Germany (mean = 8.9; SD = 11.8; CI 95% = 13.1–4.8). In this sense, the number of citations of the main countries were 341 citations for US (37.2%), 53 citations UK (5.8%); 50 citations Australia (5.5%), 48 citations for Canada (5.2%), and 37 for Germany (3.7%). The correlation between number of publications per country and number of citations was significant (Spearman’s test = 0.98; *p* < 0.01), although in other countries (Table 2), such as Spain, with a lower number of publications and citations, the correlation was lower (Spearman’s test = 0.86; *p* < 0.05). Additionally, significant differences between citations of articles published by the US and documents published by other countries were found (*p* < 0.001). The correlation test proved how documents published in the US had a positive association with more citations (Spearman’s test = 0.198; *p* < 0.001), although the coefficient was lower than expected.

**Figure 3:**
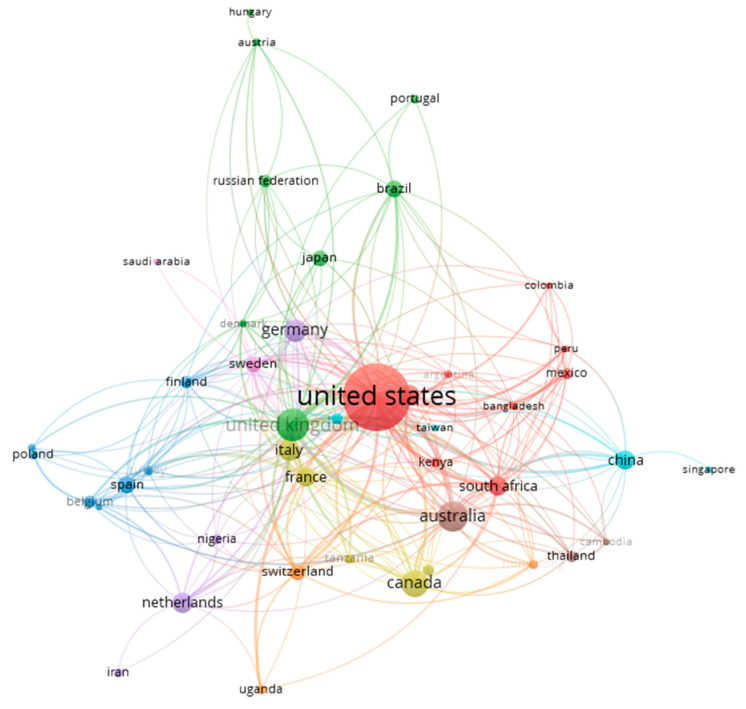
Collaboration among countries.

Furthermore, a concurrency analysis was carried out to determine possible associations between countries, showing different clusters (Figure 3). Figure 3 shows countries’ collaboration networks between 45 countries, representing the frequency of documents by the size of the circle. The figure represents the nine clusters, with the first, red cluster led by US. The red cluster, with nine countries and 279 links to countries, represents 51.3% of the collaborations between the countries. This cluster is formed by the US (presented in 408 documents), Colombia (five documents), Mexico (11 documents), India (37 documents), and South Africa (presented in 33 documents). The second cluster, in green, represents 18.1% of the collaborations between countries, led by the UK (being in 96 documents and with 36 links to other countries). This cluster was the second in number of countries (eight countries) and links, formed by the UK, Brazil (26 documents), Japan (23 documents), Russia (15 documents), Portugal (eight documents), Austria (six documents), Denmark (six documents) and Hungary (five documents). The third cluster (in blue) reflects 8.1% of the collaborations between the countries, being formed by seven countries. This cluster was led by Spain (presented in 26 documents and with 16 links to different countries), followed by Belgium (15 documents and 15 links), Finland (14 documents and 19 links), Poland (nine documents and four links), Turkey (eight documents and 13 links), Ireland (six documents and six links) and Greece (five documents and four links). The fourth cluster (yellow), represents 8.1% of the collaboration and formed by five countries, which leader Canada (presented in 65 documents and with 17 links to different countries), followed by Italy, France, Norway, and Tanzania. The following cluster in purple, represents 5.2% of the collaborations, formed by four countries with Germany as the leading country (presented in 50 documents and with 17 links to different countries), followed by Iran, Netherlands, and Nigeria. The next cluster (light blue) was formed by four countries (China, Singapore, South Korea and Singapore) was led by China (presented in 35 documents and with 15 links). The seventh (orange) and eighth (brown) clusters were formed by three countries each. The final cluster (pink) was formed by two countries, Sweden (presented in 25 documents and with 24 links to other countries) and Saudi Arabia.

### 3.3. Institution and Jounarls More Relevant in the Topic

In Table 4, the ten organizations with the highest rates of publication in the occupational safety regarding ICTs in the healthcare sector were analyzed. The University of Toronto is in the first position, with 40 publication, followed by the CDC with 31 documents. Next is the National Institute for Occupational Safety and Health, in third position (22 documents), the University of Calgary (18 documents) and followed by the University of Washington, Seattle (17 documents). These top ten institutions represented the 18.8% the publications and the 20.3% of the citation in the topic studied. Additionally, seven out of the ten institutions with higher number of publications were from US, followed by Canada, which was the fourth country with higher number of publications.

Additionally, the prominent ten journals with higher numbers of publications in this topic, according to the Scopus database, have been analyzed (Table 5). Most of the journals with the greatest number of documents published and the highest impact factors are from the US and UK, followed by Canada. Among the top ten journals, *Journal of Hospital Infection*, which is placed in fifth place, had 612 citations, representing 5.8% of the total (total citations = 10,561), followed by *Safety Science* (with 543 citations and representing 5.1%). The second journal, *American Journal of Infection Control,* had 200 citations; and the third journal had 33 citations. The ten top journals summed up 2114 citations, representing 20.0% of the citations form all the journals in the topic.

**Figure 4:**
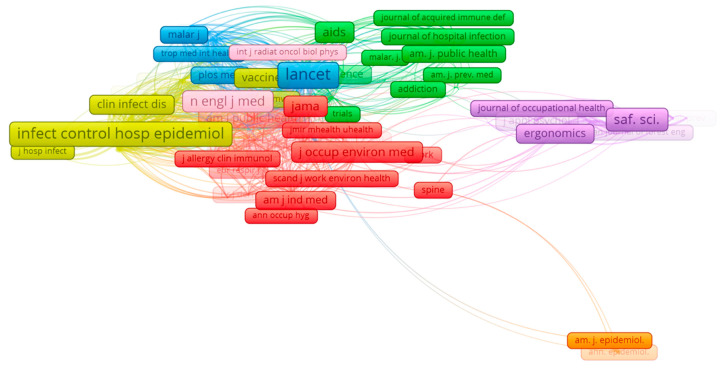
Co-citation between journals.

The analysis of the co-citations of journals also showed that these main journals (i.e., *Safety Science* or *Infection Control and Hospital Epidemiology*) were linked to other important journals (i.e., *The Lancet*) and had a major part in the clusters formed, with three (*Safety Science*, *Infection Control and Hospital Epidemiology* and *BMC Public Health*) of the seven leading journals of the cluster among the top ten (Table 5). The mapping (Figure 4) created indicated seven clusters, formed of 160 journals and 4758 links between the different journals. The first cluster (red) was formed by 51 journals, led by *Journal of Occupational and Environmental Medicine* (J. Occup. Environ. Med.), with 196 citations (Q3 and JCR of 1.64). The second cluster (green) was formed by 35 journals, led by *BMC Public Health* with 186 citations (Table 5). The third cluster (blue) was formed by 26 journals, led by *The Lancet* (Lancet) with 508 citations (Q1 and JCR of 60.39), connected to *Plos One*, which had 253 citations from other journals (Table 5). The fourth cluster (yellow) was formed by 23 journals, led by *Infection Control and Hospital Epidemiology* (Infect. Control. Hosp. Epidemiol.) with 404 citations (Table 5). The fifth cluster (purple) was formed by 179 journals, led by *Safety Science* (Saf. Sci.) with 275 citations (Table 5). The sixth cluster (pink) was formed by four journals, led by *New England Journal of Medicine* (N. Engl. J. Med.) with 225 citations (Q1 and JCR of 74.69). The final cluster (orange) was formed by two journals, led by *American Journal of Epidemiology* (N. Engl. J. Med.) with 52 citations (Q1 and JCR of 4.53).

The frequency of articles among the documents published by the top ten journals (Table 5) was between 80–90%, which matched the most frequent type of document from the data. In this sense, the most common documents published were articles (72.2%), reviews (14.9%), conference papers (8.1%) and book chapters (1.4%). The year of publication was negatively linked to the type of document, reducing the number of articles, which were the main type of documents, and increasing the number of reviews (*p* = 0.002). Furthermore, the citations per year showed significant differences between citations of articles published before 2007 (*p* < 0.001). The correlation test proved how documents published more recently had a negative association with citations (Spearman’s test = −0.385; *p* < 0.001).

### 3.4. Determination of Connections between Authors Inside the Scientific Field

**Figure 5:**
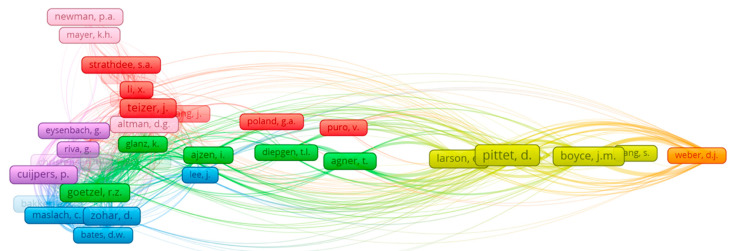
Co-citation between authors.

A further analysis was carried out based on the dominant authors in the topic, to determine co-citation among authors (Table 6 and Figure 5). Table 6 shows the scientific productions of the top five researchers focused on this subject during the last decade. Yan, L.L., from Canada, was the top author (6 documents), with an *h-index* of 48, 256 published documents and 9166 citations. Yan, L.L., started to publish in 2002 with a conference paper.

According to the *h-index* of the top five authors, Mayer, K.H. had more documents and citations, with an *h-index* of 93, 1187 documents and 45,840 citations. The second author, Brouqui, *p.* had 464 documents, 14,176 documents and an *h-index* of 60. The following author was Iavicoli, S. with 245 documents, 2354 citations and an *h-index* of 27. The last author was Canavati, S.E. with 18 documents, 240 citations and an *h-index* of 9, whose first document was published in 2011. The author with the highest *h-index* and documents was from the US, followed by France. The co-citations among authors was analyzed to determine mapping based on the references (Figure 5). The mapping identified seven clusters, with 86 items and 971 links. The first cluster (red) was formed by 21 authors, led by Teizer J., with one document and 62 citations (*h-index* of 40, 156 documents published and 5443 citations). The second cluster (green) was formed by 19 authors, led by Goetzel R. Z. with no documents and 45 citations (*h-index* of 40, 182 documents published and 6927 citations). The third cluster (blue) was formed by 15 authors, led by Zohar D. with one document and 46 citations (*h-index* of 32, 51 documents published and 7048 citations). The fourth cluster (yellow) was formed by 13 authors, led by Pittet D. with one document and 113 citations (*h-index* of 91, 589 documents published and 35,692 citations). The fifth cluster (purple) was formed by eight authors, led by Cuijpers *p*. with two documents and 55 citations (*h-index* of 108, 788 documents published and 41,935 citations). The sixth cluster (pink), was formed by seven authors, led by Altman D.G. with no documents and 40 citations (*h-index* of 218, 1064 documents published and 352,962 citations). The final cluster (orange) was formed by three authors, led by Weber D.J. with one document and 28 citations (*h-index* of 64, 443 documents published and 15,845 citations).

### 3.5. Determination of Sub-Topics Utilizing the Keywords

**Figure 6:**
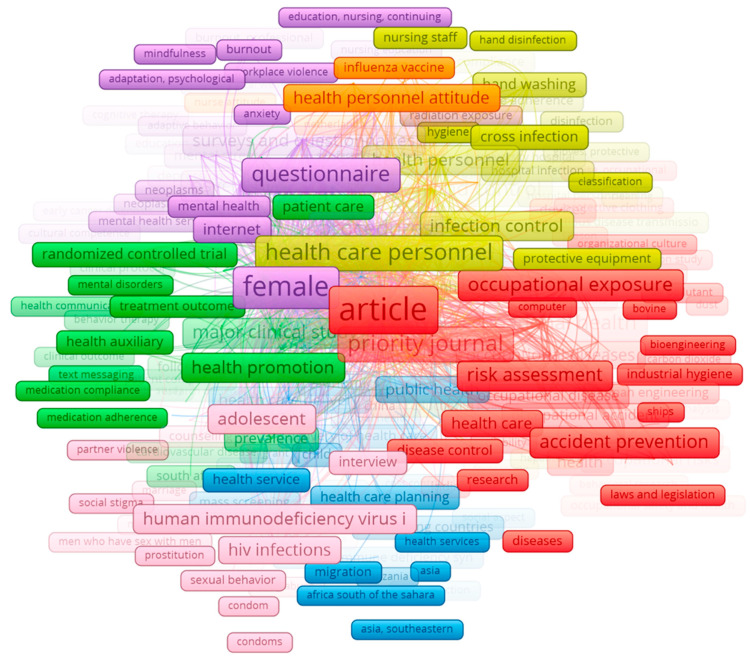
Co-occurrence of most common index terms per document. Note: the colors of the nodes indicate principal components of the data structure; the node size was scaled to the index keywords’ occurrences.

The most common topics in the data were identified using the index keywords of each document (Figure 6). The topics identified eight clusters (formed by 993 keywords with 84,876 links between them). The first and main cluster (formed by 190 keywords) focused on occupational health, prevention of diseases and accidents, and inclusion of ICTs (21.2% of the documents), such as bioengineering (red cluster). This first cluster (red) represented the main sub-topic inside the topic of occupational safety and health linked to ICTs in the healthcare sector. The second sub-topic (green), formed by 172 keywords, represented 18.3% of the sub-topics, focused on viruses and emergency pathologies that affect the global population and therefore were risks. The third cluster (in blue) was formed by 169 keywords and its frequency in the data was 16.8%. This sub-topic was based on workers’ behavior, such as smoking, and mental health, such as stress, burnout syndrome, and physiological support. The fourth cluster, in yellow, represented 16.4% of the sub-topics and was formed by 148 keywords. This cluster has as its main theme the prevention and control of infections and diseases, and correct procedures, such as handwashing and training. The fifth cluster (in purple), formed by 124 keywords and whose frequency in the data was 9.3%, concentrated on the aging of the working population and healthy factors. The six cluster (in pink, formed by 79 keywords and representing 8.2% of the sub-topics) was based on needlestick, hepatitis, devices, and prevention. The seventh cluster (in orange) was formed by 33 keywords and presented 5.1% of the nine sub-topics (clusters), and was centered on cancer, initial detention, and ultraviolet rays. The eighth cluster (in brown), was formed by 18 keywords and had a frequency among the clusters of 4.7%, and was based on insurance, preventive health services, and programs.

## 4. Discussion

This paper studied the global trend of 1021 research publications regarding ICTs in occupational safety and health of healthcare workers. The results have shown that the publication rate in this topic has increasingly grown from 1989 to 2019, and most publications were from the US and other developed countries. Although the trend of publications increased with the growth of ICTs in the healthcare sector, this trend seemed to highly increase from the beginning of the 2000s, correlating with the popularity and increased development of ICTs in the working environment. The data showed a significant difference between ICTs in OSH in 1989 compared to 2019, which could be related to the revolution of technology [48,49]. Based on previous work and the current trend linked with the fourth technological revolution, the impact of ICTs in the OSH of the healthcare sector, such as nanotechnology, sensors or virtual reality [50,51,52], might likely have a major impact over the next ten years [53].

The analysis of the data showed that most articles were published in the US and other English nations, with the main language being English, which could be related to affiliation since the main research centers focused on OSH, such as the Center for Control Disease and Prevention (CDC), are situated in US, UK or Canada [35]. Additionally, the main institutions, journals, journals and co-citations between authors and journals that published in this topic were also from these same countries (US or UK). These results could also reflect the historical background of each country regarding OSH, the integration of ICTs, the relevance of scientific production in this topic, health prevention measures, the number of inhabitants, and even the financial budget for OSH [22,54,55,56,57]. Moreover, it is interesting to note that the network of countries of this study showed how the countries with a higher frequency of publications were the leaders of each cluster. Though it is somewhat surprising that no main country was linked with other more relevant countries, they were linked to minor countries following a more aleatory structure. Nevertheless, these connections could be based on political, historical, and economic links [58,59,60,61].

Moreover, these countries where the main journals or authors carried out the research, showed associations between authors from different institutions and areas of knowledge. This interesting finding highlighted the existence of associations in the scientific community. Inside the community created, the central nucleus showed the significance of the main authors with more citations in the area, being connected to the citations, *h-index* and documents published. These results seemed to match previous papers that indicated that a connection between authors was linked to the relevance and citations of the author [62,63]

The current study also found that the most common types of publication were articles, followed by reviews. Although the type of document changed with the passing of the years, these results were contradictory to previous studies that highlighted how in medicine or public health, reviews were usually more common [64,65,66]. Although they were increasing, this reduced number of reviews could be linked to the need for previous publications and the creation of scientific knowledge in any thematic area [67,68,69].

This study’s results indicate that the topics of the studies, based on the index keywords and resulting in multiple sub-areas, were related to OSH in the healthcare sector, and some measured ICTs. The sub-topics highlighted the principal concerns and issues present at the beginning of the 21st century and that continue today, such as prevention, biological exposure, and training [66,70,71,72]. Another main topic was related to mental health, for prevention and early detection. This topic seems to reflect one of OSH’s main issues for healthcare workers that has increased after the pandemic with Covid−19 increasing sleeping issues and burnout syndrome [29,73,74]. The relevance of the different topics and supremacy of prevention might be related to its evolution and significance [75,76,77]. Nevertheless, it seems that inclusion of ICTs is more delimited to prevention and promotion of health among workers, screening systems, such as cancer [78], and new devices to prevent infections, which could be a representation of the reduced inclusion of ICTs in OSH in the healthcare sector when compared with other sectors [49,79,80,81]. Additionally, the topics seem to focus on the positive effect of ICTs in the OSH for the healthcare sector, although the European Agency indicated that the ICTs might also have negative effects for healthcare workers [79].

Another intriguing result was the citations per document and year of publication of the documents, with 2007 the breaking point. The citations per document also seemed to be related to the journal’s relevance and the thematic area. These results corroborate the findings of a great deal of previous work that indicated that the relevance of a lot of research seems to be based on the journal citation report and index [82,83,84].

These findings may be somewhat limited by the choice of keywords used for the research, by which the screening and selection of the data could have limited the number of documents and, therefore, the results and the journals. Additionally, it is important to bear in mind the possible bias in these results, based on the selection of a unique database, instead of combining two or more databases. This research focused on including close terms related to technology, OSH and healthcare workers, and excluding other terms for technology, such as “robotics” or “virtual reality”, which could have limited the results. This was primarily to avoid the possible inclusion of publications not focused on the healthcare sector in which these technologies are less present [85]. Moreover, the study of index keywords and, therefore, the topics might not represent the totality of the research carried out in the healthcare sector, as not all the keywords used were MeSH terms. Finally, the Boolean operators used, which were “OR” and “AND”, may have incorporated documents in which the main objective may differ from the objective of this paper. However, based on the sample size and the screening process, the possible error could be considered insignificant and could provoke little change in the result obtained in this research.

Overall, these findings have significant implications for the understanding of how the role of ICTs will evolve or continue in OSH for the healthcare sector, with this role focused on prevention via education, prevention, and early screening. Additionally, this scientometric analysis adds further information to the literature by elucidating the growing importance of ICTs in OSH and the future of occupational health in a sector that continues to have concerns regarding mental health or biological exposure [86,87]. These results may help to inform future investments in occupational public health, integrating new technologies and surveillance devices among workers to prevent health issues, who are traditionally a group at risk and that continues to have difficulties in modified behaviors [88,89]. The bibliometric visualizations also provide an accessible means of communicating the key findings to researchers, policymakers, and those working in public health.

## 5. Conclusions

This paper has argued that ICTs are included in the OSH for the healthcare sector, mainly in prevention and screening, although it seems that the most significant development of ICTs for this field is yet to come. In conclusion, this paper presented the global research patterns and current interests and identified the areas in which the ICTs are still missing or are less included. This research presents the main interest in the OSH related to the healthcare sector. Additionally, the results have highlighted the need for more studies focused on ICTs’ negative effect on healthcare workers. Nevertheless, more work will need to be done to determine the grade of inclusion or usage of ICTs for occupational health and safety among healthcare workers and organizations, as well as specific protocols or technological tools developed as technical safety measures.

## Figures and Tables

**Table 1:** MeSH (Medical Subject Heading) terms and description.

Mesh Terms	Description	Related Terms
Technology	The application of scientific knowledge to practical purposes in any field. It includes methods, techniques, and instrumentation.	None
Education distance	Education via communication media (correspondence, radio, television, computer networks) with little or no in-person face-to-face contact between students and teachers.	Distance Education
		Distance Learning
		Learning, Distance
		Online Learning
		Learning, Online
		Online Education
		Education, Online
		Online Educations
		Correspondence Courses
		Correspondence Course
		Course, Correspondence
Occupational health	The promotion and maintenance of physical and mental health in the work environment.	Health, Occupational
		Industrial Hygiene
		Hygiene, Industrial
		Industrial Health
		Health, Industrial
		Safety, Occupational
		Occupational Safety
		Employee Health
		Health, Employee
Health Personnel	Men and women working in the provision of health services, whether as individual practitioners or employees of health institutions and programs, whether or not professionally trained, and whether or not subject to public regulation.	Personnel, Health
		Health Care Providers
		Health Care Provider
		Provider, Health Care
		Providers, Health Care
		Healthcare Providers
		Healthcare Provider
		Provider, Healthcare
		Providers, Healthcare
		Healthcare Workers
		Healthcare Worker

**Table 2:** Number of papers and citations per country from the data.

Ranking	Country	Count of Documents	Frequency	Number of Documents with Citations	Frequency
1	United States	369	36.1%	341	37.2
2	United Kingdom	55	5.4%	53	5.8
3	Australia	51	5.0%	50	5.5
4	Canada	50	4.9%	48	5.2
5	Germany	37	3.6%	34	3.7
6	Italy	33	3.2%	32	3.5
7	China	32	3.1%	29	3.2
8	Netherlands	31	3.0%	29	3.2
9	India	28	2.7%	23	2.5
10	France	27	2.6%	21	2.3
11	Brazil	22	2.2%	19	2.1
12	South Africa	22	2.2%	18	1.9
13	Japan	21	2.1%	18	1.9
14	Spain	19	2.0%	15	1.6
15	Russia	14	1.4%	11	1.2
16	Sweden	12	1.2%	10	1.1
17	Switzerland	12	1.2%	9	1.0
18	South Korea	11	1.1%	9	1.0
19	Finland	10	1.0%	8	0.9
20	Belgium	9	0.9%	8	0.9
21	Mexico	8	0.8%	8	0.9
22	Norway	8	0.8%	7	0.8
23	Poland	8	0.8%	7	0.8
24	Portugal	7	0.7%	6	0.7
25	Others	96	9.4%	99	10.8

**Table 3:** The top ten most cited documents.

Ranking	Title	Year	Journal	Thematic Area	Study	Country	Citations
1	Environmental contamination makes an essential contribution to hospital infection	2007	Journal of Hospital Infection	Public, environmental health	Article	United States	414
2	A smartphone dongle for diagnosis of infectious diseases at the point of care	2015	Science Translational Medicine	Medicine research and experimental	Article	United States; Rwanda	219
3	Behavior change versus culture change: Divergent approaches to managing workplace safety	2005	Safety Science	Engineering, industrial	Article	United States	173
4	Percutaneous Injury, Blood Exposure, and Adherence to Standard Precautions: Are Hospital-Based Health Care Providers Still at Risk?	2003	Clinical Infectious Diseases	Infectious Diseases	Article	United States	118
5	Surveying wearable human assistive technology for life and safety critical applications: Standards, challenges and opportunities	2014	Sensors	Engineering, electrical & electronic	Review	Qatar	113
6	Modern technologies for improving cleaning and disinfection of environmental surfaces in hospitals	2016	Antimicrobial Resistance and Infection Control	Public, environmental health	Review	United States	104
7	Epidemiology of hospital sharps injuries: A 14-year prospective study in the pre-AIDS and AIDS eras	1991	American Journal of Medicine	Medicine general and internal	Article	United States	100
8	Needlestick injuries in the United States. Epidemiologic, economic, and quality of life issues	2005	AAOHN journal	Public, environmental health	Review	United States	97
9	The relationship between return on investment and quality of study methodology in workplace health promotion programs	2014	American Journal of Health Promotion	Public, environmental health	Article	Australia	85
10	Uniform: an evidence review of the microbiological significance of uniforms and uniform policy in the prevention and control of healthcare-associated infections. Report to the Department of Health (England)	2007	Journal of Hospital Infection	Public, environmental health	Review	United Kingdom	78

**Table 4:** Publications and citations by the top ten international institutions.

Affiliation	Country	Publications	Frequency	Number of Documents with Citations	Frequency
University of Toronto	Canada	40	3.9%	39	4.2%
Centers for Disease Control and Prevention	United States	31	3.0%	29	3.2%
National Institute for Occupational Safety and Health	United States	22	2.2%	22	2.4%
University of Calgary	Canada	18	1.8%	18	2.0%
University of Washington, Seattle	United States	17	1.7%	16	1.7%
University of Cape Town	South Africa	14	1.4%	14	1.5%
Johns Hopkins Bloomberg School of Public Health	United States	13	1.3%	12	1.3%
Duke University	United States	13	1.3%	13	1.4%
University of Melbourne	Australia	12	1.2%	11	1.2%
Brigham and Women’s Hospital	United States	12	1.2%	12	1.3%

**Table 5:** Quartile and Journal Citation Report (JCR) of ten principal journals.

Source	Documents Published	Quartile Score	JCR (2019)	Documents (2019)	Citable Items (2019)	Frequency of Articles	Country
*Safety Science*	22	Q1	4.11	461	512	90.82%	Netherlands
*American Journal of Infection Control*	19	Q2	2.29	345	303	88.45%	United States
*BMC Public Health*	13	Q1	2.51	1764	1741	94.77%	United Kingdom
*International Journal of Environmental Research and Public Health*	12	Q1	2.47	5186	2843	90.82%	United States
*Journal of Hospital Infection*	12	Q1	3.27	272	202	87.13%	United Kingdom
*BMJ Open*	11	Q2	2.50	4303	3887	85.13%	United Kingdom
*Infection Control and Hospital Epidemiology*	11	Q2	2.94	367	236	88.56%	United States
*Journal of Medical Internet Research*	11	Q1	5.03	1895	643	84.45%	Canada
*Malaria Journal*	10	Q1	2.63	450	431	93.50%	United Kingdom
*Plos One*	10	Q2	2.74	2.92	11,244	97.31%	United States

**Table 6:** Progress of the top five authors’ works during the last decade.

Year	Yan, L.L.	Brouqui, *p*.	Iavicoli, S.	Mayer, K.H.	Canavati, S.E.	Total Documents
2009	0	0	0	0	0	0
2010	0	0	1	0	0	1
2011	0	0	0	0	0	0
2012	0	0	0	0	0	0
2013	0	0	0	0	0	0
2014	1	1	0	0	0	2
2015	1	1	1	0	0	3
2016	0	1	0	1	3	5
2017	1	1	0	1	0	3
2018	0	0	0	0	0	0
2019	3	0	1	1	0	5
Total Documents	6	4	3	3	3	19
